# Head teachers’ opinions on the future of school education conditioned by emergency remote teaching

**DOI:** 10.1007/s10639-021-10600-5

**Published:** 2021-06-05

**Authors:** Katarzyna Potyrała, Nataliia Demeshkant, Karolina Czerwiec, Beata Jancarz-Łanczkowska, Łukasz Tomczyk

**Affiliations:** Institute of Educational Sciences, Pedagogical University of Cracow, 2 Podchorazych Str, 30-084 Cracow, Poland

**Keywords:** Education, Emergency Remote Teaching, School as learning organization, Pandemic, Head teachers

## Abstract

The study explores the school transformation process as evidenced by the opinions of head teachers. The main goal of the research was to present a content analysis study of the Polish educational environment on the basis of primary and secondary head teachers’ views on the risks and perspectives brought by the global Covid-2019 lockdown. The conceptual framework was based on the theoretical perspective (the cognitive and affective processes in multimedia learning, the theory of motivation, and goal setting) as well as the model of the school as a learning organization and the assumptions of *Emergency Remote Teaching.* The categorized interviews with the head teachers were conducted using a categorized interview questionnaire and the respondents considered various categories problems within educational practice related to the functioning of schools during the pandemic. The selection of study participants was deliberate using the snowball sampling method, 18 head teachers participated in this study. The research conducted allowed the specification of the different areas of influence of *Emergency Remote Teaching* on the transformation of the school as a learning organization (e.g. the functioning of the school in mutual internal and external network cooperation, the dissemination and extending of communication areas using information technologies, the strengthening of the network interaction through information technologies, and other areas). The paper contains numerous recommendations that can improve the school's functioning in the future, based on the experience gained during *Emergency Remote Teaching*. These experiences can accelerate the organizational and didactic development of the school as a learning organization.

## Introduction

Observations of the pedagogical environment reveal that teachers largely base their teaching concepts on many years of professional practice as well as on their own educational experience gained as students (Hargreaves & Fullan, 2020). Nowadays the significant impact of the pandemic in terms of teaching and learning is apparent. The pandemic created conditions that challenged traditional teaching strategies, the quality of feedback between teachers and students, collaboration between teachers and parents, and the entire educational community (Hargreaves & Fullan, 2020; Tomczyk et al., 2020). The current educational circumstances are so unique that some authors propose to define this new situation as ‘crisis learning’ (Tejedor et al., 2020). During the COVID-19 pandemic, most governments around the world temporarily closed the schools. The students were forced to quickly review their metacognitive strategies and to develop their own remote learning style. The educational changes that have been made around the world as the result of the COVID-19 pandemic have revealed problems with equitable access to technology (often called the ‘digital divide’) around the world (UNESCO, 2020; Zhong, 2020). The situation with the transition to the delivery of educational courses via the Internet, known as *Emergency Remote Teaching* (Milligan, 2020; Hodges et al., 2020), caused distress of teachers who had little or no previous experience with online methods and techniques of teaching.

Similarly, there were many schools which lacked an established plan and infrastructure for transitioning their entire student life to the online modality (Gorham & Ogata, 2020). Repeatedly, attention is drawn to the problems of students resulting from the prolonged state of physical isolation from peers, teachers, extended family and other real-world social networks (Zhang et al., 2020). Meanwhile, little is known about the long-term effects of isolation and forced social policies as well as education and interactions mediated by ICT tools on the mental health of children and adolescents, as well as the future of school education as a whole. It is predicted that the rapid global shift towards online education will further exacerbate inequality in educational attainment for students around the world (Petrie et al., 2020).

*The aim of this research* is to present a content analysis study of the Polish educational environment on the basis of primary and secondary head teachers’ views on the risks and perspectives brought by the global Covid-2019 lockdown.

Due to the fact that the competences of the head teachers include, among other, the formation of schemes of cooperation between all the actors of the school community and managing the learning environment (Hu, 2017; Mustamin, 2012; Singh & Miah, 2020), they form a group whose opinion may affect the future of education after the pandemic.

The views of head teachers are important because they play a key role in the operationalization of policy, strategy, and vision.

## Conceptual background

The conceptual background of the research was focused on teaching and learning processes as well as school responsibilities in the new social reality after the pandemic. The starting point was the teaching process supported with IT tools, which for several years has been successfully adapted in school practice, most often in the form of hybrid education or as teaching tools to make the teaching process more attractive. Typically, e-learning has been applied in the case of open universities, and m-learning was treated only as a variation on standard lessons. The new educational reality caused by the pandemic has turned this system upside down, making e-learning the main form of educational activity at all levels of education. Globally, using IT in some school subjects allowed more interactive learning, and students were more motivated and interested in using technologies such as augmented reality (AR) and virtual reality (VR). Ultimately, IT made a significant contribution to improving the students' learning outcomes of some school subjects during the pandemic, which contributed significantly to the school's success as an institution educating for the future (Iwanaga et al., 2021; Yang et al., 2020; Abdel-Hameed, 2020). However, e-learning was also often associated with problems resulting from the lack of interactivity, the simple transmission of knowledge from the computer screen, and the exclusion effect due to the lack of access to ICT tools (Lehmann, 2020). Previous studies highlighted the major barriers for online learning. The authors underlined more deep feelings of a lack of community, technical problems, and difficulties in understanding instructional goals (Song et al., 2004; Martin, 2020).

Furthermore, there is a low level of preparedness among students concerning the use of Learning Management Systems (Parkes et al., 2014). In addition, there have been cases of cognitive anxiety accompanied, as in previous global pandemics (Qiu et al., 2017), by crises related to health concerns, negative health, economic and even national security consequences around the world.

Based on the theoretical perspective of the Cognitive Theory of Multimedia Learning, CTML (Park et al., 2015; Mayer, 2005), Bandura's theory of motivation (1986, 1997), Locke and Latham's theory of goal setting (1990) and the model of the school as a learning organization (Potyrala, 2008), the research was planned, and based on interviews with head teachers, the effectiveness of teaching and organizational work of the school during the pandemic was assessed in the context of its further development as a learning organization (Table 1).

**Table 1:** Review of the theories related to the research

Theory	Components	Practical Considerations
Cognitive Theory of Multimedia Learning (CTML)	active information processing, affective and metacognitive mediation, taking into account individual differences between students (foreknowledge, cognitive styles and skills) that affect the effectiveness of the methods and media used	The use of ICT in education goes beyond the media infrastructure and is associated with the didactically and educatively justified use of IT in supporting the individual and organizational development of the school community (Mayer, 2005**;** Parkes et al., 2014). Planning the didactic and educational process in the use of ICT in teaching-learning processes results from the core curriculum for general education and the school's duties, for which the teachers and the head teachers are responsible. During the COVID-19 pandemic, the educational process and school responsibilities are carried out online, this proving an important context for the research.The new circumstances were related to a new approach to the learning and teaching process.
Theory of motivation	self-efficacy, level of motivation and performance	An employee's sense of ability affects their perception, motivation and performance (Bandura, 1997). The theory of the research assumes the perspective that modern concepts of education management recognize leadership as a key feature of successful school activity and have an impact on the effectiveness of the organization in individual and institutional terms, especially in the current crisis caused by the pandemic. A transformational leader motivates employees and supports their creativity.
Theory of goal setting	the relationship of goals with the effectiveness of activities, types and functions of goals in a learning organization, setting group goals	Goals direct attention and effort to activities essential to the goal at the expense of non-essential activities. Goals can motivate students, teachers and school heads to use knowledge and skills, or they can motivate them to seek new knowledge. This occurs when people face new, complex tasks (Locke & Latham, 2006). This situation has been experienced during the pandemic by teachers and school principals while teaching.
Model of school as a learning organization	conceptual approach to curriculum, social cooperation, motivation, building social dialogue about the objectives of education	The transformation of the school must be accepted socially; information about changes is important, as is gathering opinions about innovative ideas. Generating a favorable atmosphere is necessary for undertaking such challenges (Potyrala, 2008). The main components in the transformation of the school environment during the pandemic generally consisted of what is known as *Emergency Remote Teaching*. At the same time, this period represents the beginning of a change in thinking about the future of formal education.

Assuming that the head teacher supports teachers in their efforts during the pandemic, the role of the head teacher as a school leader is to facilitate the continuation of education for all community members through well-structured and closely-monitored distance learning.

**Fig. 1:**
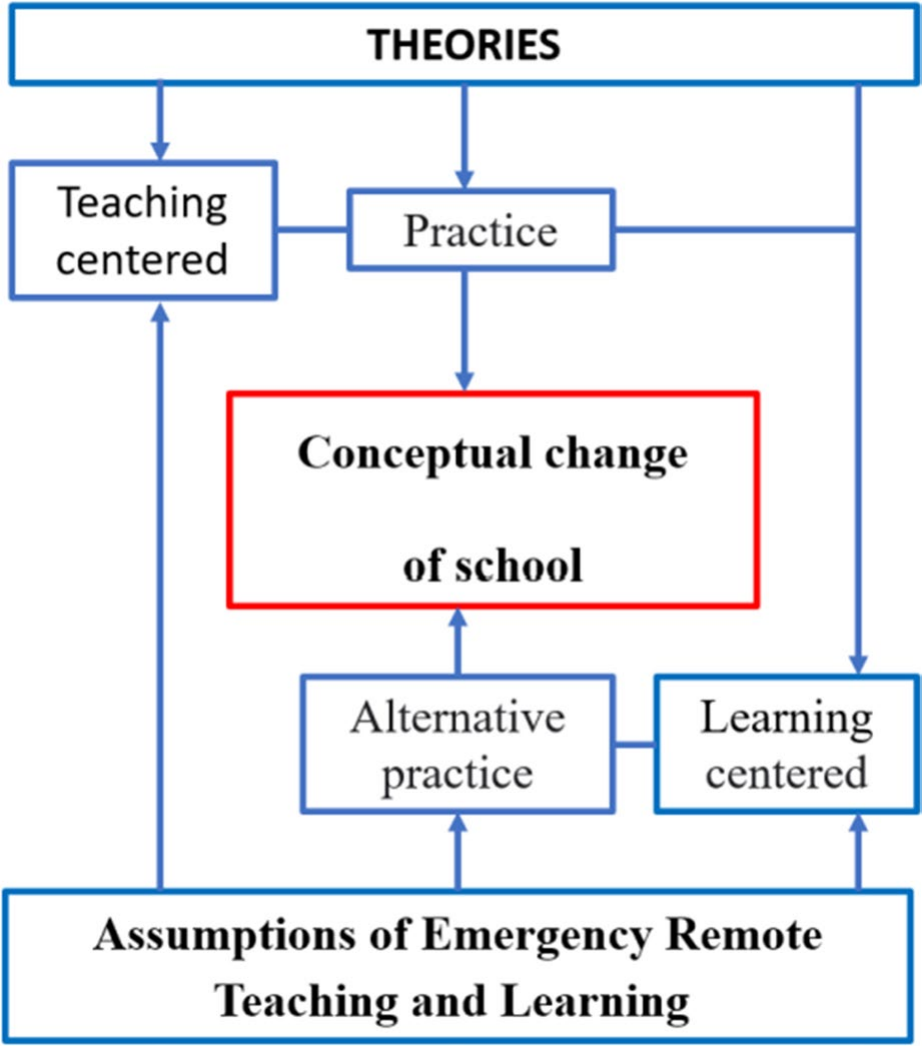
Conceptual framework (CF). Source: authors on the basis of a variety of conceptual or theoretical perspectives

Qualitative research on the prospects of the school and the role of officials responsible for the readiness to implement a range of new social practices was conducted during the flu pandemic in the summer of 2017 by Faherty et al. (2019). These were focus interviews on teleconferences and webinars with school management from across the United States. Topics stemming from the focus group protocol domains as well as unexpected emerging issues were identified and characterized. They were as follows: the need for effective communication with management, the importance of partnership for the acceptance of the situation, the impact of social distance on increasing anxiety, ensuring the safety of students, the synergistic effect of educational practices, challenges related to the enforcement of educational results, lack of funds for school nurses, different views on the role of schools in public health, the need for education and community involvement to ensure consistent implementation of different practices, the need for shared decision making, and the tension between standardizing public health guidance and adapting to local contexts. Despite the methodology limitations demonstrated by Faherty et al. (2019), the adopted research methods and tools were seen to be effective and inspired the authors of this article to use them further. The categories of practice considered by the respondents turned out to be an interesting proposition: they were so-called normal in-school practices (implemented within the normal school schedule) and limited-time practices (implemented under a revised schedule during the pandemic). As this manuscript focuses on the future of the school in the face of its transformation during the COVID-19 pandemic, the limitations and possible facilitation of specific practices in the future are outside the scope of this article. Research conducted by Lunenburg (2011) has shown that the most effective team performance occurs when the goals of the organization are achieved in conjunction with feedback, in an atmosphere of commitment and the acceptance of decisions. Head teachers and moderators have a motivational influence on achieving goals, and achieving group goals is as important as setting individual goals (Lunenburg, 2011). Almost every modern organization has some form of goals setting (e.g. management by objectives, benchmarking, etc.) that give strategic direction to the organization, and schools are no different in this regard. Therefore, it was assumed that values and goals are very important determinants of behavior, and the role of the school principal is to set goals that motivate teachers to work, especially in the non-standard situation resulting from work in the pandemic. Members of the ‘learning organization’ must be aware that the ultimate goal requires the gradation of difficulties, and its achievement is a long-term, multi-stage process. The individual stages are the successive stages of development. Hence, the school as a ‘learning organization’ should be adapted to the general model of such an institution, but due to its specific goals it should be subject to slightly different processes. The way the school is perceived by the environment, the students' parents, and institutions cooperating with the school, is of importance here (Potyrala, 2008). Emergency remote teaching is defined (according to Hodges et al., 2020) as a sudden interim shift of instructional delivery to an online delivery mode as a result of an immense disaster, in contrast to those online courses which are initially planned and designed to be delivered virtually (Hodges et al., 2020). Figure 1 shows the conceptual framework for the planned research.

## Methodology

### Research aims and question

In formulating the research aims, we have gone beyond what we found in the form of research reports and theoretical considerations in the available literature of the research subject. We added our research curiosity and desire to explore the topic due to its topicality and the new contexts that emerged from education during the pandemic. Placing the research in specific social realities and drawing the authors' attention to the factors conditioning the improvement of school responsibilities with the use of ICT in the context of social change (the changing realities of the 21st century, including the educational reality) make the work an original social study with educational implications. The following ***research aims*** were formulated: 1/ the identification of the challenges caused by the pandemic for changes in the school as a learning organization; 2/ the interrogation of the head teachers about various aspects of school work during the pandemic; 3/ the study of the head teachers’ opinions on the perspectives of ICT-assisted school after the pandemic.

The following research question has been formulated: *How has Emergency Remote Teaching affected the transformation of the school as a learning organization?* The research question posed in this way includes detailed questions about the development of the school as a learning organization during a pandemic and the perspectives of changes in the functioning of the school after the pandemic COVID-19.

### Participants

In the school year 2020/2021, interviews were conducted with head teachers of public primary and secondary schools. The participants constituted a deliberately heterogeneous sample representing all of the different types of schools in the *Malopolska* Province in South Poland. 18 head teachers participated in the study, including 8 head teachers of primary schools, 2 head teachers of secondary schools, 6 head teachers of schools arranged in groups of schools (primary and secondary school, or nursery and primary school), 1 head teacher of a nursery school and 1 head of a Youth Cultural Center. The majority of the schools are located in the city (15), the remainder (3) in the countryside. The criterion for choosing the schools was related to the opinion about the school in the educational environment and its leaders who are willing to cooperate with the social environment. Detailed characteristics of the schools whose head teachers participated in the interviews is presented in Table 2.

**Table 2:** Characteristics of the schools whose heads were interviewed

Type of school	Characteristic of the school
primary school head teachers (8 in total)	7 urban state schools; 1 rural private school number of teachers: from 30 to 75 number of class units: from 9 to 37 number of students: from 160 to 393
secondary school head teachers (2 in total)	2 urban state schools number of teachers: from 76 to 108 number of class units: from 29 to 33 number of students: from 800 to 830
head teachers of schools arranged in groups of schools (6 total)	4 urban state schools; 2 rural schools (1 state and 1 private) number of teachers: from 25 to 60 number of class units: from 9 to 26 number of students: from 150 to 650
head of the Youth Cultural Center (1)	state institution of after school education: number of teachers: 11 number of class units: 11 number of students: 150
head teacher of a nursery school (1)	rural local nursery school, number of teachers: 12 number of class units: 5 number of students: 125

The deliberate selection of study participants was performed by searching the websites of schools in the *Malopolska* Province and using *snowball sampling* - a method of non-random sampling consisting of recruiting participants through other participants (Castillo, 2009).

### Research design and tool

The interviews were conducted using a structured protocol (categorized interview questionnaire) by 6 moderators trained in qualitative methods. The respondents considered various categories of educational practice (Table 3), but were also eager to discuss problems related to the functioning of their school during the pandemic. All of the categories were assigned to specific research aims. Each research aim was explained by five different categories. The categories were revised frequently by four researchers to increase the validity of the research findings.

**Table 3:** Categories of educational practice discussed by the participants according to the research aims

Research aims	Types of categories
1. Identification of challenges caused by the pandemic for changes in the school as a learning organization.	1.1 Network cooperation between teachers and school management 1.2 Online and offline relationships 1.3 Sanitary restrictions during Covid-19 1.4 Teachers' competences in the field of remote teaching and educational activities in crisis situations 1.5 Students' competences in the field of distance learning
2. Interrogation of the head teachers about various aspects of school work during the pandemic	2.1 Interpersonal communication via ICT 2.2 Activity of participation in remote classes 2.3 Enforcing the results of remote education 2.4 Pedagogical and psychological support for teachers and students during the pandemic 2.5 Setting goals by the head teacher as a remedy for stress and a sense of threat
3. Study of head teachers’ opinions on the future of ICT-assisted school after the pandemic	3.1 Limitations and possibilities of ICT in remote teaching currently and in the future 3.2 Responsibility of students for self-education 3.3 Services of external companies and institutions in the field of training in the use of ICT in teaching 3.4 Autonomy of the school and the role of the head teacher in the decisions specific to the school environment 3.5 The authority of the teacher

### Data analysis

After the moderators interviewed the head teachers, the recordings were transcribed, and then the different categories of educational practices mentioned in the interviews were identified and coded. When coding the opinions of head teachers, categories were assigned to words or phrases that represented important (and recurring) topics within each answer.

**Fig. 2:**
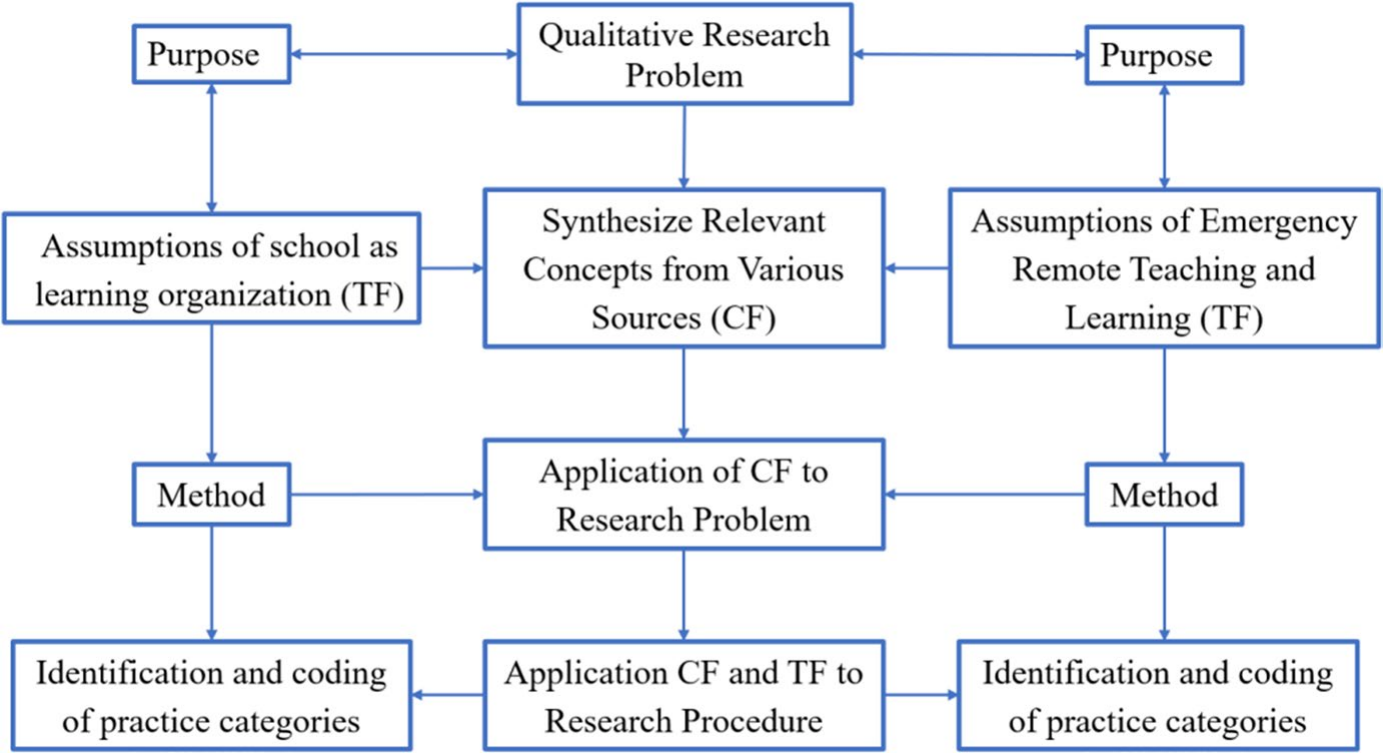
Links between the Theoretical Framework (TF) and Conceptual Framework (CF) and the study procedure

In vivo codes were used during the interviews, the respondents presented the scope of the organization's activities in their own words. In vivo codes allowed the recipients to familiarize themselves with the results of research from the perspective of the respondents, often bringing out something not obvious, surprising and fresh from the subject of research. Hence, they were later used, for example, to organize a research report. These codes were not identical to the other categories that had an overarching, analytical and theoretical character. By ‘coding’ and ‘decoding’ were understood the processes of marking and organizing qualitative data in order to identify different topics and the relationships between them. Coding qualitative research to find common topics and concepts is part of the thematic analysis, which is part of qualitative data analysis. Thematic analysis extracts topics from the text by analyzing words and sentence structure. In this case, concept-based coding was used in accordance with the guidelines of Vaughn and Turner (2016), Stuckey (2015) and Gibbs (2007), which means that the analysis started with a predefined set of codes and then assigned these to new qualitative data. These codes were related to the categories considered to be thematic areas of the analysis. Figure 2 shows the relationship between the Theoretical Framework (TF) and the Conceptual Framework (CF) and the research procedure.

The theoretical assumptions of school as a learning organization, contain references to the theory of leadership and the theoretical assumptions of Emergency Remote Teaching and Learning are related to the theory of motivation. Hence, Fig. 2 does not list all the components of the theoretical framework.

## Study results

### Identification of topics and their interrelationships

**Fig. 3:**
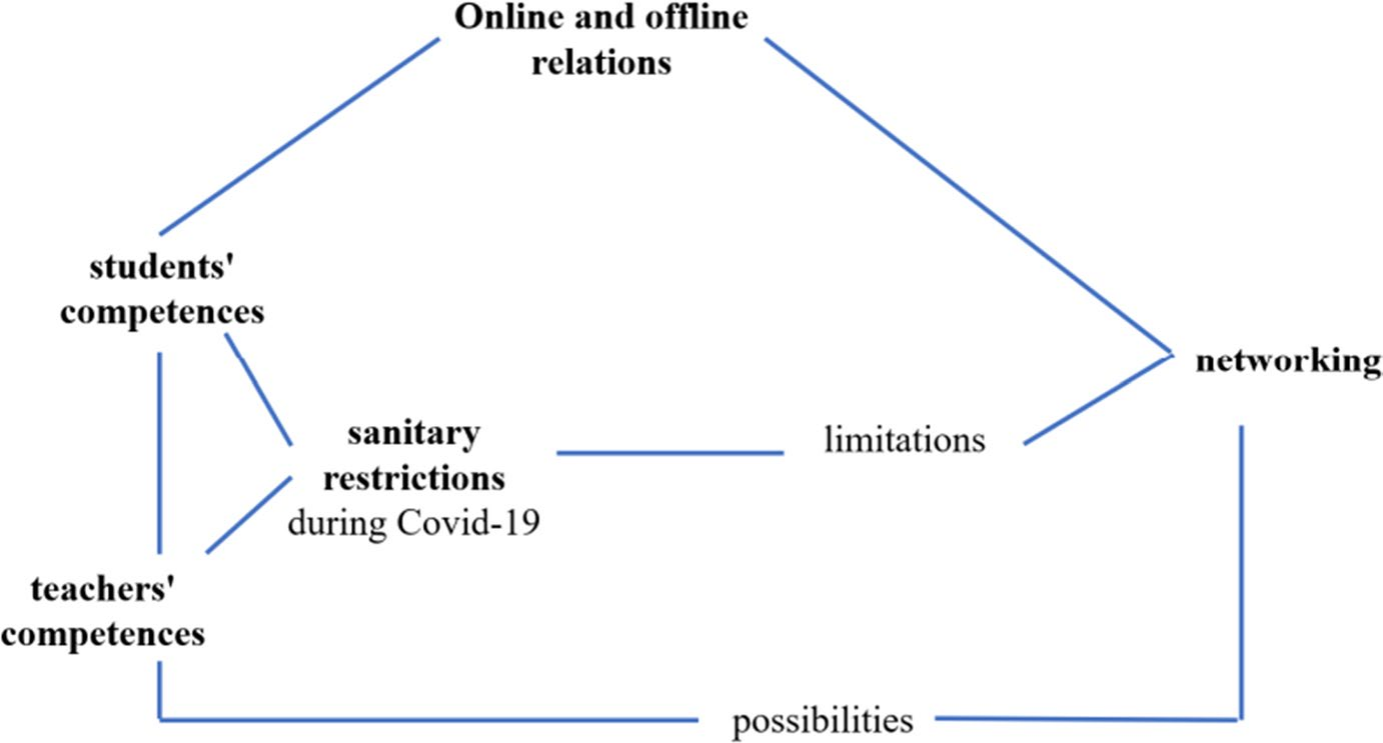
Links between categories related to the main challenges for the organization of school work during the pandemic

**Fig. 4:**
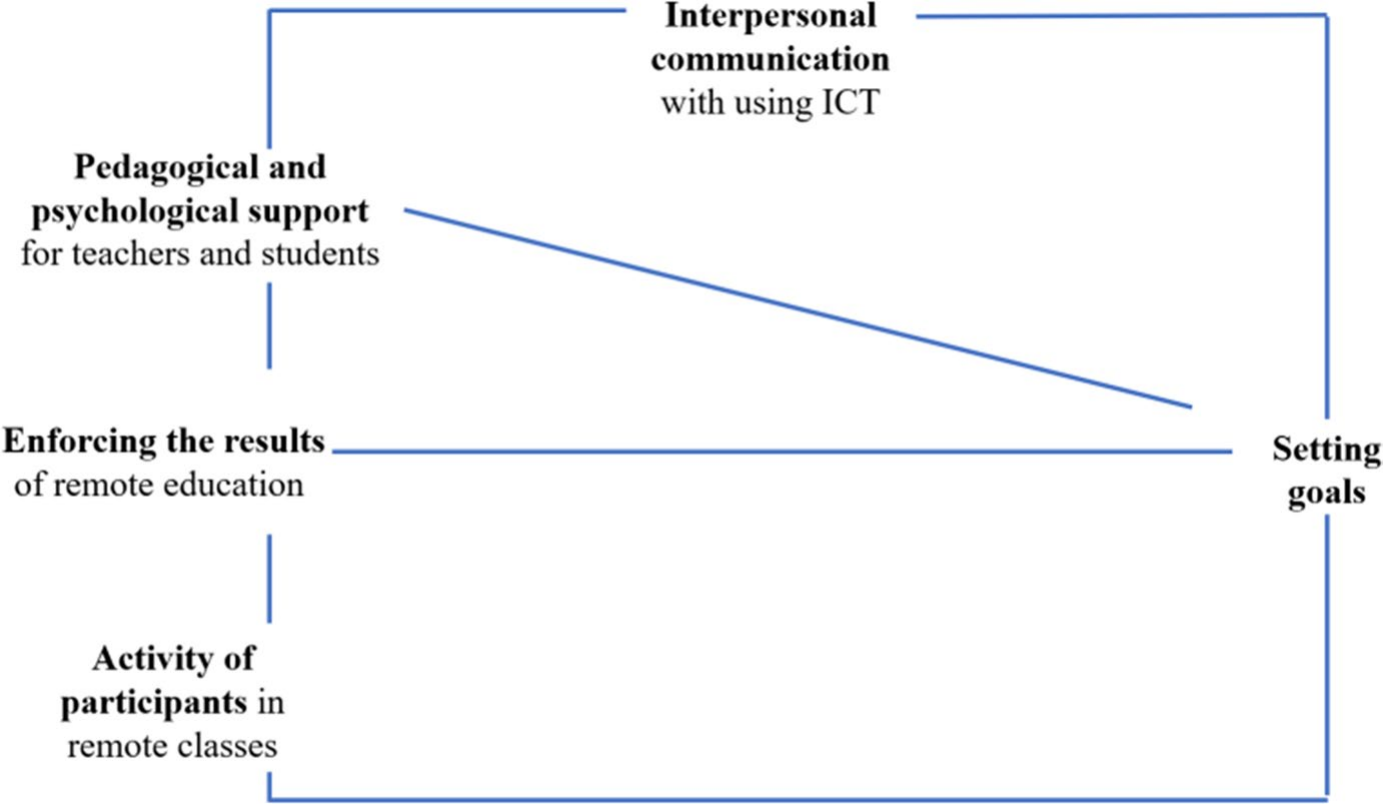
Links between categories related to different aspects of the functioning of the school in the context of goal setting during ERT

**Fig. 5:**
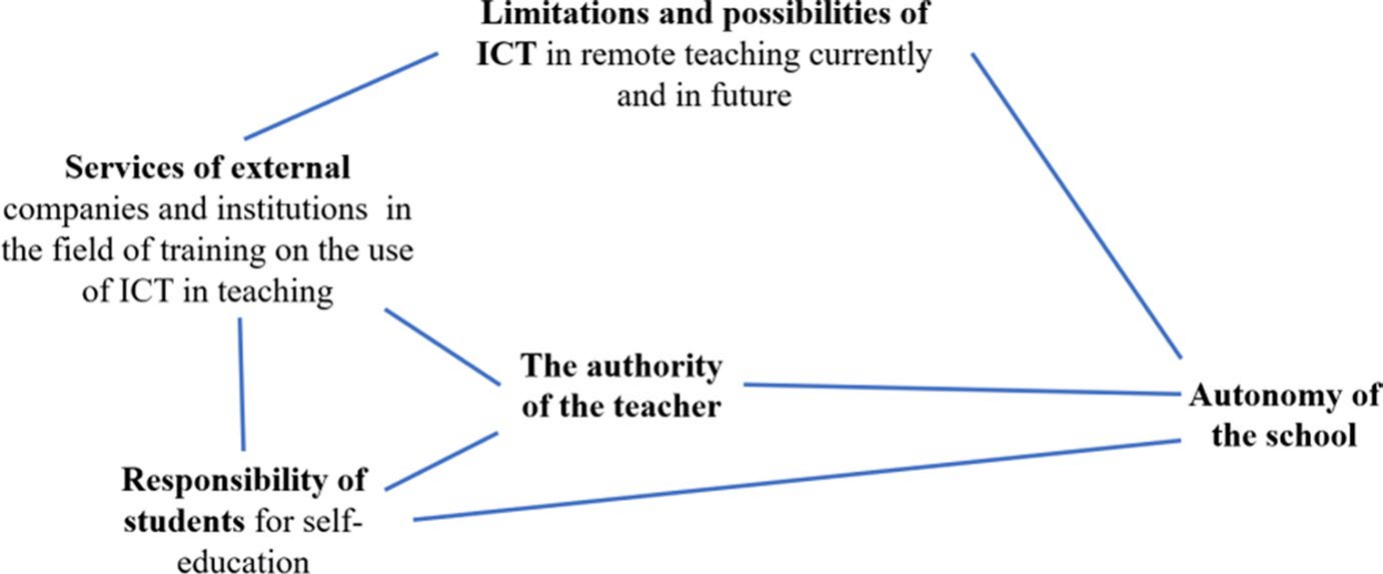
Links between categories regarding the future of ICT-assisted school after the pandemic

The thematic analysis of the interviews in accordance with the established categories allowed the arrangement of mutual relationships and the links that existed between them, and this led to the singling out of the basic categories according to the research objectives of the study (Figs. 3, 4, 5).

The most frequently discussed topics were issues related to the school's teaching staff coping with the challenges caused by the pandemic. The best solutions were sought, inter alia, through the development of a cooperation network between teachers and school management (category 1.1). In their replies, the head teachers emphasized that the period of the pandemic and distance learning is also a period of dynamic organizational changes in the school. One of the head teachers said that ‘*the school had to be reinvented’*. The dynamic situation forced cooperation between all the members of the organization, that being the school. Only through close cooperation, as emphasized by the respondents, could the situation be saved and the teaching process allowed to continue. All the respondents emphasized that they owe their success to team activities. The group activities, according to the head teachers, allowed for the efficient organization of distance learning, but also gave enormous psychological support to teachers, especially those who were less capable of dealing with communication technologies.

‘The most important thing is that we acted as a group, we supported each other. We talked a lot. Everyone can find support in the teachers’ room’.

‘One of the teachers, who is better at the application, trained the others in such trial classes, trial lessons, so such mutual help was the most important’.

‘We try to pass on good practices to each other. It is also important to me that the school should be such a community as this one’.

In the interviews, the head teachers also emphasized how important it was for them to cooperate with other school heads. They called each other, exchanged their observations, and suggested solutions that had been adopted in their schools. Among the head teachers of the schools in which the research was conducted, there was a close professional network, about which one of the heads said: “*I was happy to accept the decision of such a network that was created between the head teachers*”. The heads and teachers collaborate to monitor what is happening with the students during the pandemic and are in constant contact with the parents. Many issues were solved at the beginning of the pandemic thanks to this ongoing cooperation. Clear procedures were developed jointly by the school heads and teachers and then explained to the parents. In some responses, the head teachers indicated the need for close cooperation with the Ministry of Education and the Superintendent’s Office, especially in times of crisis, when mutual agreement on decisions was particularly needed.

‘It would be perfect if there was a platform where one could meet representatives of the government, be it local or state, and in some way present their proposals, because such sensible ideas are born during brainstorming’.

A category frequently mentioned in the interviews concerned online and offline relationships (category 1.2). The head teachers emphasized that all the members of the school community would like to return to the offline form of education because the direct teacher-student relationship is of such great importance. The lack of offline contact raises concerns among teachers about the effects of implementing the teaching material and verifying the students' achievements. A teacher conducting an online lesson in a large class is not able to see all the students, even if they have cameras on, does not recognize their emotional states, and cannot ask about the students’ reactions and emotions in lieu of direct observation.

It was not unusual that the response of the principals often alluded to the sanitary restrictions connected with COVID-19 (category 1.3), especially in situations where the need to reconcile “*Sanepid”* Service recommendations regarding the school during the pandemic conflicted with the desire of students and parents to experience school in the traditional mode.

The head teachers considered it a great challenge to reconcile sanitary restrictions with the provision of individual consultations for students in need, as well as technical and methodological support for teachers.

A very important issue that appeared in all the interviews was the teachers' competences in remote didactics and educational activities in crisis situations (category 1.4). As mentioned earlier, the pandemic situation and the transition of entire school communities exclusively to distance learning was an unexpected situation. The period March-June 2020 was especially difficult for teachers. However, one head teacher emphasizes that ‘*teachers are aware that this is the only system to be implemented at any given time and that it cannot be different. Therefore, everyone must perform their tasks, because there is no other option*’. Teachers with different levels of competence in the use of ICT in education found themselves in a new professional situation at the same time. The respondents report on how difficult this professional transition was for them. Sometimes even the most talented teachers had problems with remote education. However, with joint support, the situation was soon stabilized.

‘Some teachers are great in the classroom, but not necessarily good at remote teaching’.

‘The teachers had to learn a lot, support each other and the stress in this case was very beneficial’.

All the interviewed head teachers emphasized that in the period from March to October 2020, and especially in the post-holiday period, when it was known that the return to distance teaching was inevitable, the teachers made a huge leap in the development of their digital competences. Technical barriers were overcome and the focus turned to the methodology of remote classes.

‘I see it as a training, teachers no longer ask how to turn on the camera. They have only problems with more complex tools that can be used in teaching online. Teachers see many positives in using technology. More and more often we do not pay attention to technical matters anymore, we focus on what to do to make the classes even more interesting’.

The head teachers emphasized the difference between the way they worked in spring 2020, when teachers mainly sent messages to students via the electronic journal and only some connected online with their students, and what it is like now when all the teachers deliver lessons online. However, two head teachers pointed out that there were still groups of teachers for whom remote teaching was extremely problematic and that they were tired of this form of teaching. Another respondent sees the potential for teacher development in the current situation, claiming that ‘*teachers will draw conclusions and will want to learn, they will want to use information technology to improve their teaching methods under normal conditions, not only during the pandemic’.*

A powerful challenge to the school due to the pandemic turned out to be the competence of students in the field of distance learning (category 1.5). In the interviews of head teachers, there were often reflections on students' skills with using IT tools in remote lessons, and problems with discipline or performing tasks during remote classes. Head teachers pointed to the students’ lack of ability to work independently, and the growing reluctance of some students to engage in online lessons: ‘*students often work without cameras, and there is no legal way to change this’.*

The main challenges in the organization of the schools during the pandemic were mostly caused by the need to comply with sanitary restrictions, which disrupted online and offline connections and networking. At the same time, there were possibilities for the development of teachers 'and students' digital competences, which in turn helped overcome the limitations.

The next group of topics raised by the head teachers concerned various aspects of the school's functioning during the pandemic (the second research goal). Among others, they pointed to the role of interpersonal communication using ICT (category 2.1). The head teachers emphasized that communication platforms had also become a meeting place for teachers and students and provided an opportunity to create specific support groups.

‘[Informal groups in Microsoft Teams] is an idea for them to survive the stress of remote learning" .

One of the respondents pointed to the time-consuming nature of remote contact.

‘Remote doesn't mean faster. The exchange of information is not direct, it requires writing an email, so formulating sentences, someone has to receive the information, write a reply, send it back. A 3 minute reaction often changes to a matter of 3 days’.

In addition, head teachers emphasized that teachers have difficulties maintaining contact with students with disabilities or dysfunctions, as well as with students with difficult home conditions or a difficult family situation. In such situations, with parental consent, the students were able to participate in remote classes but from the school premises.

An important issue for the organization of the efficient functioning of the school in the distance learning mode is the activity of participants in remote classes (category 2.2). The respondents pointed to the need to undertake more activities to motivate students to learn than in the case of teaching in a traditional environment, and to technical limitations that prevent the effective motivation of students.

‘As a head teacher, I am terrified by the issue of preparing students for external exams, in such a remote version, preparing them is a very difficult thing’.

‘The next important thing is how to motivate students to study independently. It is common to avoid calls, turn off cameras, avoid participating in lessons’.

The head teachers emphasized that although their teachers do not have difficulties in communicating with students, the students are aware that the supervision of their work by the teachers is not as thorough as it would be in the offline classroom. This is unfortunately visible in the relatively low activity of students during remote lessons.

The subject of enforcing the effects of distance learning (category 2.3) was also broached by the head teachers during the interviews. It is difficult for teachers to solve the problem of assessment, and it is highly frustrating for teachers to be aware of the students' unrestricted use of textbooks, online resources, and even third party help when testing knowledge. Therefore, the teachers specify their expectations of help in solving this issue.

“I would expect and I think that teachers too, tools, I do not know how prepared, maybe by IT specialists, which would help us to fairly verify the knowledge and skills of students”.

According to the head teachers, educational units under the Ministry of Education should develop such criteria and evaluation of such examination papers, in a way that is consistent with the realities of remote teaching.

An exceptionally important topic that appeared in every interview was the pedagogical and psychological support of teachers and students during the pandemic (category 2.4). The head teachers reported that in their schools, school educators and psychologists are on online duty. They create contact schedules with students and parents. In turn, the class teachers conduct educational lessons on topics related to the current situation, as well as arrange individual consultations with students. The head teachers and educators are aware that the separation from peers does not have a positive effect on the students and that there will be psychological and pedagogical problems after the pandemic. A favorable solution for overcoming stress conditions caused by the lack of contact with peers involves hybrid forms of school functioning, when the school was always available to those students willing to attend. The students could go to meet in small groups with their teachers, use school equipment or access to the Internet, all while respecting the principles of the sanitary regime. Some head teachers emphasized that ‘*the fact of conducting classes in the normal mode is for children mental reinforcement*”, “*we have time for children from dysfunctional families and here they can find such a safe haven and support as opposed to institutions that are quite remote on teaching’*.

The subject of proper management, the leading function of the administrative team for the efficient functioning of the school, especially in crisis situations, setting goals by the head teacher as a remedy for stress and a sense of threat (category 2.5) was all present in the interviews. Organizational solutions were heterogeneous, as the head teachers were given a lot of autonomy from the ministry, supervisory and managing organs, which in most cases met with approval. In this diversity of adopted solutions, certain common elements can be noticed, e.g. cyclical meetings, consulting the solutions adopted with teachers and parents, and referring, in the case of unclear situations, to the rules and principles introduced by the orders of the school principals. The whole picture of the changes described shows the image of the headmaster as a person who plays a key role in this crisis situation.

‘The repealed provisions had to be replaced with other solutions. To my great surprise in the first decree introducing the rules of online learning, nothing had been properly changed until June ’.

One of the respondents pointed out that the rules and procedures developed earlier, before the pandemic, for another crisis situation were very useful at her school.

‘At school, we have developed procedures for dealing with crisis situations. When I started school, it was in a deep crisis. The first moment of the pandemic was a shock. In March, you had to find yourself in a completely new environment. And the procedures developed for crisis situations helped us in this. We have set out guidelines for action. I wrote to the teachers every morning keeping them in the group, making sure no one in the group fell out. Later, we trained very intensively. We had a trainer who walked us through one of the platforms’.

Support in the pandemic situation was something the head teachers provided each other with through intensive cooperation between the closest institutions.

"I work with head teachers and management of other institutions in the city, with community and city community centers, and we support each other because the rules change so quickly that we meet and discuss how best to do it."

Figure 4 presents the links between categories related to different aspects of the functioning of the school in the context of goal setting during ERT in the head teachers’ opinions.

Setting goals was the central issue which allowed to ensure the functioning of the school in various aspects, including through pedagogical and psychological support for teachers and students, enforcing the results of remote education, interpersonal communication with using ICT and activity of students in remote classes.

The third group of topics in the head teachers’ interviews concerned the future of ICT-assisted school after the pandemic (the third research goal). All of the responses related to the limits and opportunities of ICT in the current and future remote school work (category 3.1). All respondents agree that the period of distance learning brought about by the pandemic has caused irreversible changes in education. The majority of respondents indicate that these changes are profound and good, that it is something that was actually expected.

‘I think we've all gone through a mental change, all the teachers and students and parents, and I don't think that can go back. This change in my opinion is positive. Education had to move on’.

Teachers recognise that ICT tools are helpful in many areas of education and, above all, they improve communication between teachers.

‘It seems to me that in classroom teaching it would be possible to make good use of what we already know, what the students can do. For example, adding MS Teams to complete projects, connect tasks. In the case of teachers' councils meetings, we connect online and it turns out that we can all quickly connect and get things done’.

‘We will definitely stick to some of the solutions we have developed at the moment. These are all kinds of online applications that can make the class more attractive’.

At the same time, the respondents are aware of the need to conduct research in the field of distance learning, the conclusions of which would contribute to the development of the methodology of remote teaching.

‘For now, e-learning requires research on larger populations, but I have a feeling that we are facing a paradigm shift in learning and teaching’.

According to the head teachers, online learning presents many challenges connected with learners’ issues, educators’ issues, and content issues. In addition to technical problems e.g. a lack of reliable broadband Internet, limited Internet data plans for students relying on mobile devices, or students who do not have a desktop/laptop computer, the directors in their replies indicated the lack of constant interpersonal communication with the use of ICT in remote school learning, and delayed comments and feedback from the teacher and/or student when assessing student knowledge. In addition, head teachers pointed out again a number of ICT opportunities for remote work, especially for administrative matters (remote meetings of the teachers' board, evaluation and implementation of educational projects at school).

‘A lot of statistical tools have emerged that teachers can use to evaluate their own work’.

An important issue regarding the future of the school after the pandemic, according to most head teachers, was the responsibility of students to engage in self-education (category 3.2). There are expectations of changes in students’ attitudes to learning and an increased sense of responsibility for their own development. One of the head teachers also expressed the hope that *‘‘parents and students will understand that everybody learns for himself/herself and that is the most important thing’.*

An important area of consideration given the experiences at the start of the pandemic is the services of companies and external institutions in the field of training in the use of ICT for education (category 3.3). The dynamics of the development of the pandemic and the rapid closure of schools due to the increasing number of cases became a breakthrough moment for education. All the respondents emphasize this in the interviews.

‘We were forced at a rapid pace to use IT tools in our lives, something changed in education 100%’. ‘We previously applied for funding for distance learning projects for students who have individual tuition and cannot contact the teacher for health reasons. These projects found no support. In general, remote education was ‘mocked’ – ‘give the students computers and they will start to play’. In the spring, there was no other choice. Either we switch to distance learning, or there will be no teaching at all’.

Initially, in the period from March to June 2020, despite the numerous technical problems caused by the lack of a uniform educational platform for e-learning, the limitations in the availability of high-speed Internet connections and, at a most foundational level, the insufficient competences of teachers and students in the use of ICT in teaching and learning, attempts were made to teach remotely. The cooperation of head teachers with city offices was generally smooth. Schools received the necessary ICT equipment, and access to the Microsoft Teams platform. Training on the platform was also funded by the city. The state gave schools laptops for those children who had problems with access to appropriate equipment.

One topic that relates to the optimism of head teachers for the future of school education is the autonomy of the school and of the head teacher’s functions in making decisions specific to the school environment (category 3.4). Most of them positively received the transfer by supervisory institutions and authorities of a high degree of autonomy in the area of decisions concerning the organizational solutions adopted at their school.

‘The more autonomy we have, the more we can do. Autonomy made it possible to conduct exams, for example. We only have some knowledge inside the school and it is impossible to regulate certain matters from the top’.

Some head teachers admitted, however, that it would be easier for them to make decisions if certain things were determined centrally (e.g. by the health department, the education department, or school management) and consulted with the school running organ. In their opinion, each school is different and has its own specificity, but certain patterns and examples of good practice in this area should be provided from above.

The teacher's authority was also an important issue (category 3.5). The head teachers drew attention to the behavior of parents, which at the beginning of the pandemic was not fully understood. Parents participated in remote lessons, prompting children or commenting on the individual activities of the teacher and students. This proved a discomfort for both the children and their teachers. Over time, the parents appear to have noticed that the teachers are trying to conduct online lessons reliably and have thus abandoned these practices. Thanks to the pandemic situation, parents generally appreciated the factual and didactic competence of the teachers.

Figure 5 presents the links between the categories regarding the future of ICT-assisted school after the pandemic according to the head teachers’ opinions.

Autonomy of the school is directly correlated with the authority of the teachers and limitations and possibilities of ICT in remote teaching. According to the head teachers, in order to ensure the sustainable functioning of the school, especially in crisis situations, the school should be able to decide on its own regarding the organization of school education, the use of external technical support, which in turn will contribute to the growth of the responsibility of students for self-education.

### Identification of the positive and negative effects of ERT on school education in the opinion of the head teachers

The analysis of the interviews allowed the positive and negative effects of ERT on the functioning of the school education system to be distinguished (Table 4).

**Table 4:** The positive and negative effects of ERT on the functioning of the school education system

The positive effects	The negative effects
New cooperation and support networks	ICT-mediated relationships
Teachers take up of activity in the crisis situation of the pandemic	Declarative activity of students, ICT users
Teachers’ task-oriented approach to achieving goals	Psycho-emotional problems of students as a consequence of isolation
Diagnosis of students’ problems and attempts at pedagogical interventions supported by external institutions	No individual teachers’ and students’ responsibility for solving team tasks
Specifying institutional goals by the head teachers and strict executive supervision with particular emphasis on educational goals	Individual restrictions on access to open knowledge resources and the use of applications enabling collaboration for the entire community
Increased (step by step) sense of responsibility for learning outcomes and self-development in students	Poor academic performance reflected in students' final grades in the school year covered by distance learning
Dynamic development of teachers' competences in the field of remote teaching didactics	Health problems of students and teachers resulting from working at the computer (e.g. problems with eyesight)
Improving the school equipment with respect to teaching tools (e.g. computers, laptops, tablets, software)	Some teachers leaving the profession
Making state authorities aware of the need to equip schools with ICT tools and the positive aspects of using online education	Lowering the standard of education
Parents noticing and appreciating the effort made by teachers in educating students	Lack of school distance learning standards
Increase in digital and technological competences of students, teachers, and parents	

The analysis of the positive and negative effects listed in Table 4 for the school learning system shows that ERT contributed to the increase in digital and technological competences of teachers and students, strengthening the ICT facilities of schools, as well as improving cooperation between the main entities of the school educational process - teachers, students, parents. As one of the respondents stated:

‘A crisis is such a phenomenon which causes the system to crumble in order to put it back together, but at a higher level. It was like that here too - it hurt, but something good will come out of it’.

## Discussion

The research analysis suggests that the majority of Polish schools did not have any difficulty in switching to online teaching, as participation in the class was easier, mainly through the possibilities offered by technology, but also because of the means of communication between the teachers and the students. This conclusion is in accordance with other studies on distance education and e-learning (Panitsides & Karapistola, 2020; Pavlis-Korres, 2017). A decisive advantage presented by school distance learning and favoring the overcoming of stress caused by the general restriction of social contact in the school context, was the permanent communication and cooperation between the teachers as well as between teachers and students, parents, and the local government. This result differs from the results of previous online education studies that have identified a lack of communication and cooperation, as well as the general restriction of social contact in the academic context (Karalis & Raikou, 2020). In addition, the head teachers’ responses show that through online teaching new skills related to distance learning are developed by the teachers as well as by school students and parents. In terms of teaching content, the majority consider this to be covered by distance learning as well, but a significant percentage of respondents do not agree with this assessment, arguing that face to face learning covers the subject being taught to a greater extent. These results are in line with previous findings, according to which it is seen as challenging to develop content which not only covers the curriculum but also engages the students (Kebritchi et al., 2017). Educational technology researchers have stressed that the main task of *Emergency Remote Teaching (ERT)* is not to re-create a robust educational ecosystem but rather to provide temporary access to instruction and instructional support in a manner that is quick to set up and is reliably available during an emergency or crisis (Hodges et al., 2020).

The head teachers highlighted that there is a lack of standards for quality, quality control, the development of e-resources, and e-content delivery. This problem needs to be tackled immediately so that everyone can enjoy the benefits of quality education via e-learning (Cojocariu et al., 2014). Other authors have expressed the opinion that while advantages exist in the adoption of online learning during the crisis, the development and enhancement of online courses should also be a key concern when looking to the future (Affouneh et al., 2020).

This is not as easy as it seems; a considerable amount of investment is needed for getting the devices and equipment, maintaining the equipment, training the human resources, and developing the online content. Therefore, an effective and efficient educational system needs to be developed to impart education via the online mode (Dhawan, 2020).

Everyone involved in this sudden school transformation into a learning organization must recognize that crises and disasters also disrupt the lives of students and teachers. ERT must be effected with the understanding that switching to online education is unlikely to be a priority for everyone involved (Hodges et al., 2020). The respondents had different approaches to the changes that took place and not all of them took up the challenge presented by the new way of managing their school, expecting from the authorities guidelines on how the school should work. Meanwhile, there could be more asynchronous activities than synchronous ones and this does not contradict the idea of transforming the school as a learning organization. In the context of the future of school education, the transition from ERT to more pedagogical-driven distance learning will be greatly interesting to monitor (Schultz & DeMers, 2020). Many head teachers expect the further development of remote teaching, and support for school development and ICT learning strategies (Eger et al., 2020; Al Ghazo et al., 2020).

The unexpected circumstances related to the pandemic forced head teachers to adopt a new way of managing the school and enforcing results. The initial outcomes were generally unsatisfactory for the students, teachers, and parents, but many effective solutions were developed over time. This article describes the positive and negative aspects of the experience and presents best educational practices. This type of research has so far been undertaken rarely and only on the basis of subject didactics (Schultz & DeMers, 2020; Fedeli, 2012), and the results of research among head teachers obtained by the authors of this article shed new light on the future of ICT-supported schools after the pandemic.

## Conclusions

**Fig. 6:**
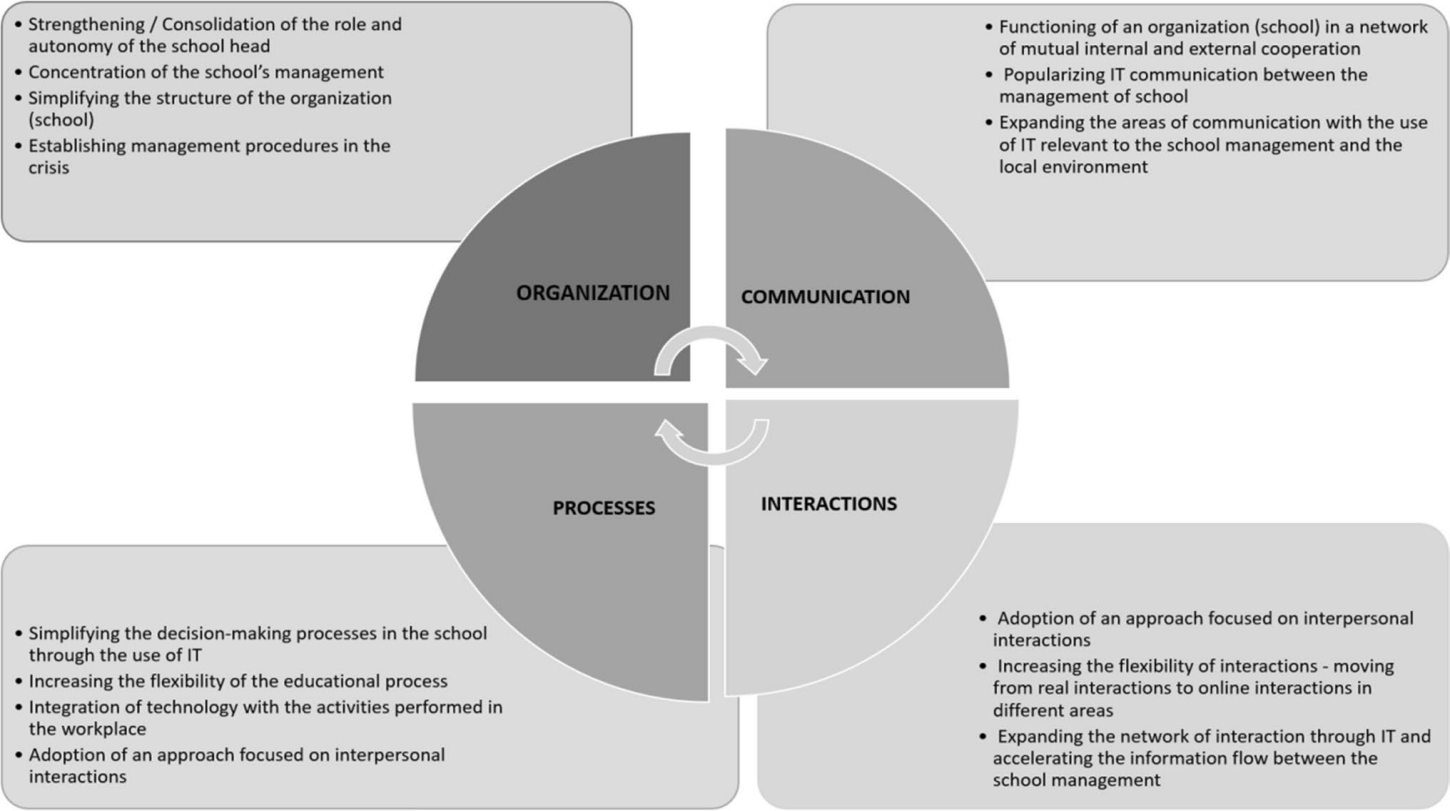
Main areas of influence of Emergency Remote Teaching on the transformation of the school as a learning organization

There are four main areas in the school function which were directly touched during pandemic Covid 19: organization, communication, processes and interactions. Each of them requires special attention from school principals. The research allowed us to define the ways of advising principals in each of these areas (Fig. 6).

The study results showed that communication was the most important component in school functioning for efficient Emergency Remote Teaching. This element includes: the functioning of the school in a mutual internal and external network of cooperation, popularising IT communication between the management of the school, expanding the areas of communication with the use of IT relevant to the school management and the local environment. All together it influenced the strengthening of the network interactions through IT, which was defined as the second element of the school sustainable functioning during Covid lockdown. This element includes: adoption of an approach focused on interpersonal interactions, increasing the flexibility of interactions - moving from real interactions to online interactions in different areas, expanding the network of interaction through IT and accelerating the information flow between the school management.

Embracing the challenges of internal and external school cooperation and interactions positively influence on the processes as the third element of the school functioning during Emergency Remote Teaching, which provides: simplifying the decision-making processes in the school through the use of IT, increasing the flexibility of the educational process, integration of technology with the activities performed in the workplace, adoption of an approach focused on interpersonal interactions.

These processes were crucially important for the school organisation as a whole through strengthening the school head, concentration of the school's management, simplifying the structure of the school, establishing management procedures in the crisis.

In connection with the implementation of the research aims and the research problem the following conclusions were formulated:

1. The main challenges caused by the pandemic have been identified for changes to the school as a learning organization. The period of school closures caused by the COVID pandemic contributed to the strengthening of teamwork in schools and the institutional or spontaneous networking of teachers. Working in teams within the institution, as well as inter-institutional cooperation and the mutual assistance of teachers and head teachers, were of key importance for the rapid development of competences in the use of IT in remote teaching and for the mental condition of employees in this crisis situation. All management in the educational process recognise the fundamental differences between human interactions taking place in the real world and interactions in the virtual world. The recognition of these differences stimulated the need to understand the technical possibilities of interaction on various remote communication platforms. Remote communication platforms have become a place of informal meetings for students and teachers, which allows the minimization of the effects of social isolation. Difficulties in communication with students with dysfunctions via IT were solved by enabling offline contact within the school in compliance with the relevant sanitary rules. The emergency online teaching situation has accelerated the progress of the development of digital literacy for teachers and students.
2. Head teachers’ opinions on various aspects of the school's functioning during the pandemic were recognized and should be taken into account when developing a strategy for the functioning of schools in the future. They stated that interpersonal communication using ICT and the activity of participants in distance learning are the basic conditions for the success of distance learning. However, verifying the effects of education through the use of ICT is still difficult. The protracted distance learning situation mobilized head teachers who began to pursue goals to overcome online learning difficulties as well as to improve remote communication for all members of the school community.

The pandemic forced head teachers to seek new technologies and remote forms of work. Establishing close cooperation during the pandemic is a manifestation of the development of civilization, and the diversity of cooperation between head teachers, teachers, parents, students and institutions is one of the guarantors of recovery from crisis situations such as the one currently being faced.

Head teachers’ opinions on the future of ICT-assisted schools after the pandemic were examined. It was found that the quality of self-education activities during the pandemic largely depends on the level of awareness of all the actors in the educational process. Therefore, there is an urgent need to provide adequate support for students who are least prepared for distance learning. The solutions are effective online instruction, and the development of diagnostic tests and other tools that reflect the students’ actual knowledge. The combination of traditional education with distance learning increases the level of interest of students and their activity during classes. This creates the conditions for the further development of hybrid teaching and learning in school with the support of external entities in the field of ICT.

Taking into account the development of the school of the future, a very important element within the entire system is the autonomy of the school management in making decisions in crisis situations and the support of this autonomy by school supervisory institutions, including in the use of ICT.

The findings of this study have to be considered, however, in the light of some limitations. Since this is a subjective study where head teachers’ statements provided the data, the researchers cannot entirely rule out limitations in the collection and analysis of subjective data measuring the perspectives of head teachers regarding selected elements of emergency remote teaching. Another limitation of this study is the selection bias of the study participants due to the limited access to the geographical region. The interviews were conducted with purposely heterogeneous samples of head teachers in the schools of Lesser Poland, thereby constituting a sample bias since these respondents may not truly be a good representation of schools across the country. To address these limitations, future studies should be conducted with a qualitative research approach to form a mixed method study, as doing so will ensure that there is confirmation of the head teacher's perspective about the research subjects, thereby minimizing the sample bias.
